# A novel approach for direct detection of the *IGH::CRLF2* gene fusion by fluorescent in situ hybridization

**DOI:** 10.1186/s13039-023-00652-2

**Published:** 2023-08-13

**Authors:** Rosa María González-Arreola, Adriana García-Romero, María Teresa Magaña-Torres, Juan Ramón González-García

**Affiliations:** 1 Doctorado en Genética Humana, Centro Universitario de Ciencias de la Salud, Universidad de Guadalajara, Guadalajara, Jalisco Mexico; 2 División de Genética, Centro de Investigación Biomédica de Occidente, Instituto Mexicano del Seguro Social, CIBO-IMSS, Sierra Mojada #800, Colonia Independencia, CP 44340 Guadalajara, Jalisco Mexico

**Keywords:** Acute lymphoblastic leukemia, *IGH::CRLF2* gene fusion, BCR-ABL1-like, Fluorescent in situ hybridization, Break-apart probes, Gene fusions

## Abstract

**Background:**

High expression of the Cytokine Receptor-Like Factor 2 (*CRLF2*) gene has been observed in patients with acute lymphoblastic leukemia BCR-ABL1-like subtype. Currently, there is no commercial system available for the direct detection of the *IGH::CRLF2* fusion by fluorescent in situ hybridization (FISH), as there are for many other leukemia-related gene fusions. In an effort to verify the *IGH::CRLF2* fusion, some researchers prepare home-grown FISH probes from bacterial artificial chromosome clones flanking the *IGH* and *CRLF2* genes, which is the best alternative to confirm the fusion, however difficult to reproduce in most cytogenetic laboratories.

**Results:**

For the direct observation of the *IGH::CRLF2* gene fusion we designed a methodological approach requiring the two commercially available IGH and CRLF2 break-apart probes.

**Conclusions:**

Our methodological approach allows direct visualization of the *IGH::CRLF2* gene fusion and has the potential to be used for identification of other gene fusions.

## Background

High expression of the Cytokine Receptor-Like Factor 2 (*CRLF2*) gene has been observed in subsets of patients with T or B-cell acute lymphoblastic leukemia (ALL), especially in those with BCR-ABL1-like subtype of B-cell precursor ALL. The main molecular mechanisms leading to high *CRLF2* expression are either an intra-chromosomal deletion causing the *P2RY8::CRLF2* fusion or one of the chromosomal translocations t(X;14)(p22;q32) and t(Y;14)(p11;q32), which produce the *IGH::CRLF2* gene fusion [1, 2]. These translocations cannot be detected by traditional cytogenetic banding techniques.

Since its role in lymphoid transformation in B-cell precursor ALL was described [1, 2], detecting *IGH::CRLF2* gene fusion in most cytogenetics laboratories has posed a great challenge. Currently, there is no commercial system available for the direct detection of the *IGH::CRLF2* fusion by fluorescent in situ hybridization (FISH), as there are for many other leukemia-related gene fusions. Most cytogenetic laboratories perform one or two independent FISH studies with CRLF2 break-apart or IGH break-apart probes (CRLF2-BA or IGH-BA) to establish the presence of the *IGH::CRLF2* fusion [1–15]; however, this approach, although strongly suggestive, does not actually confirm the fusion. This is why, in an effort to verify the *IGH::CRLF2* fusion, some researchers carry out their FISH studies through break-apart probes on metaphase cells processed either by the trypsin-Giemsa method or by additional FISH studies with centromeric or painting probes, in order to identify the chromosome acceptor of the translocated signal (i.e. the der(14) for FISH with the CRLF2-BA probe, or the der(X)/der(Y) for FISH with the IGH-BA probe) [1, 3, 6, 10, 11]. The main drawback of this strategy is that, although it allows for unequivocal identification of the t(X;14)(p22;q32) and t(Y;14)(p11;q32) translocations, it not only does not prove the fusion but its process is affected by other variables, such as the low or null mitotic index of cell preparations, the poor chromosomal morphology most leukemic cells show, the heavily skewed ratio of abnormal to normal metaphase cells, often in favor of normal ones, among others. The best alternative researchers have found to confirm the *IGH::CRLF2* fusion is to do a FISH with a single-fusion extra-signal probe mixture prepared by themselves from bacterial artificial chromosome (BAC) clones flanking the *CRLF2* and covering the *IGH* genes [2, 3, 8, 12, 13]. The manufacture of home-grown probes is a long, laborious, and costly process; in addition, it demands proper knowledge and infrastructure to carry out the procedures related to the culturing and processing of BAC clones, and their fluorescent marking, which is essential for their application in FISH experiments. Due to all these factors, probe manufacturing cannot be performed by most cytogenetic laboratories.

## Results

**Fig. 1:**
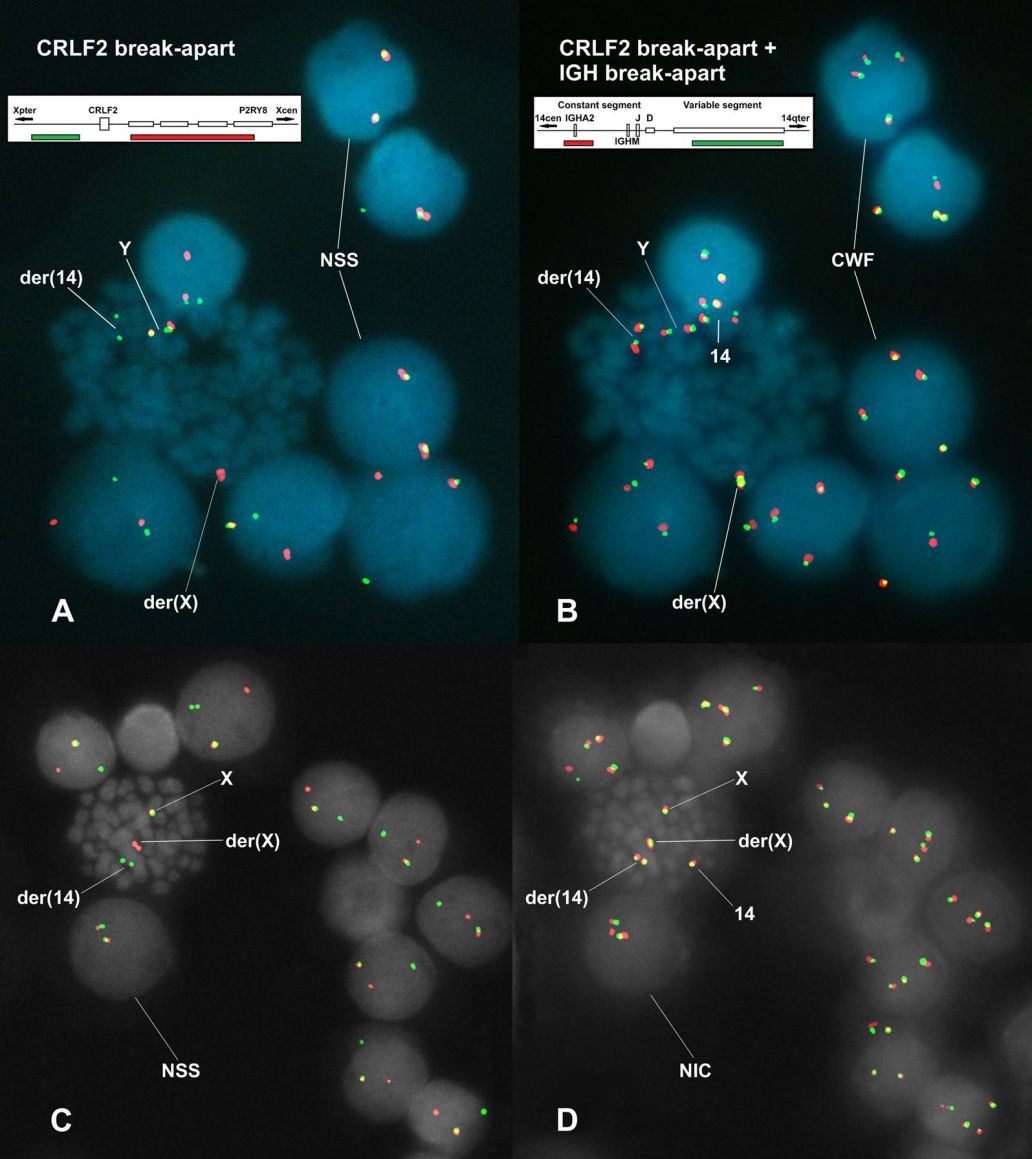
Serial FISH studies performed with CRLF2-BA and IGH-BA probes, which are schematically represented in inserts. **A** and **C**: FISH performed with the CRLF2-BA probe. Almost all cells display the separation of one pair of red/green signals, which is the hallmark of the presence of a *CRLF2* rearrangement. However, in A there are two cells and in C one cell showing non-separated signals (NSS), suggesting they lack a *CRLF2* rearrangement. **B** and **D**: FISH performed by mixing both the CRLF2-BA and IGH-BA probes. Comparisons of A vs. B and C vs. D allowed us to observe that the separated proximal red signal of *CRLF2,* in A and C, appears connected with the separated distal green signal of *IGH* in B and D; whereas, the separated distal green signals of *CRLF2* observed in A and C appear, in images B and D, connected with the separated proximal red signal of *IGH*. Remarkably, the red/green NSS, observed in two cells in A, remained unchanged in B, appearing two new pairs of connected red/green signals ─which correspond to the two normal copies of *IGH*—meaning that they are cells without the *IGH::CRLF2* fusion (CWF). *NSS* Non-separated signals; *CWF* Cells without the *IGH::CRLF2* fusion; *NIC* Non-informative cell

With the methodological approach here presented we made the following observations. The separated *CRLF2* signals observed in cells from Fig. 1A and C show a perfect coupling with the separated *IGH* signals revealed with the FISH study using the CRLF2-BA/IGH-BA probe mix (Fig. 1B and D). We applied this methodological approach in 6 patients with ALL (4 women and 2 men), selecting 100 informative cells per patient and, through this procedure, the *IGH::CRLF2* fusion was directly demonstrated in all (100%) the informative cells.

## Discussion

Currently, the strategy preferred by some researchers for the study of ALL is, firstly, to carry out studies focused on the detection of overexpression of the *CRLF2* gene by flow cytometry or RT-PCR [1–3, 6–8, 13–15]; our methodological approach could facilitate the confirmation of the *IGH::CRLF2* fusion as the cause for such overexpression.

Moreover, other neoplasia-associated gene fusions, for which there is no commercially available two-gene dual-color probe, could be addressed with the approach here presented by us to confirm their existence. Some of these gene fusions are *IGH::BCL6, IGH::TRA, IGH::TRD, IGH::MYC, IGK::MYC, IGK::BCL2, IGK::BCL6, IGL::BCL2, IGL::BCL6, IGL::MYC, TRA::MYC, RUNX1::MECOM, FUS::ERG, FUS::DDIT3,* and *EWSR1::DDIT3,* among others.

In addition, our method could be applied in those chromosomal rearrangements (translocations or inversions) where a known gene from a derivative chromosome is broken (for example the *IGH* gene), but where the partner gene from the other derivative chromosome is rarely affected (as fusions of the *IGH* gene with *CEBPA, CEBPB, CEBPD, CEBPE, CEBPG* genes), or even unknown. For these instances, the combination of a commercial red/green probe (as IGH-BA probe) with another probe prepared from BAC clones and labeled in blue/aqua spectrum, could be an excellent approach to clearly identify such fusions.

## Conclusions

Our methodological approach allows direct visualization of the *IGH::CRLF2* fusion and has the potential to be used for identification of other gene fusions.

## Methods

For the confirmation of the *IGH::CRLF2* fusion we designed a methodological strategy requiring the two commercially available IGH-BA and CRLF2-BA probes (CytoCell^®^; catalogue numbers LPH 014 and RU-LPH 085, respectively), which is described in the following steps.

1. First and preferably, cases potentially positive for the *IGH::CRLF2* fusion should be studied by FISH with the CRLF2-BA probe (Fig. 1A and C) (note: it is preferable to start with the CRLF2-BA probe because there are many gene fusions affecting the *IGH* gene; while for *CRLF2*, outside the fusions generated by the intra-chromosomal deletion (which can also be detected by this probe) only *IGH* is the reported partner (although Harvey et al., [3] reported an unknown fusion, their case 9906_113)). This FISH study is performed following the supplier's instructions.
2. During the analysis, it is important to photograph and record the coordinates of those microscopic fields selected by the presence of metaphase and/or interphase cells showing an evident separation of the proximal-red and distal-green *CRLF2* signals; these cells were considered as informative cells.
3. After recording several microscopic areas, the glass cover-slip is removed by capillarity in 2xSSC 0.05% FISH-washing solution.
4. Immediately, a second FISH is performed with a CRLF2-BA/IGH-BA probe mix adjusting the recommended supplier´s final volume with equal amounts of each probe.
5. The procedures for dehydration, hybridization, post-hybridization washing and counterstaining with DAPI (4′,6-diamidino-2-phenylindole) are the same as those performed in the first FISH experiment, i.e., following the supplier's recommendations.
6. Locate the previously recorded microscopic fields and reanalyze them with emphasis on the informative cells.

## Data Availability

Data generated and analyzed during this study are included in this article. Further inquiries can be directed to the corresponding author.

## Abbreviations

BA: Break-apart
BAC: Bacterial artificial chromosome
CRLF2: Cytokine Receptor-Like Factor 2 gene
CWF: Cells without the *IGH::CRLF2* fusion
IGH: Immunoglobulin heavy chain gene
NSS: Non-separated signals
